# Elements of Medicine

**Published:** 1859-09

**Authors:** 


ART. VIII.—*Elements of Medicine; a Compendious View of Pathology and Therapeutics; or, the History and Treatment of Diseases.* By SAMUEL HENRY DICKSON, M.D., Professor of the Practice of Physic in the Jefferson Medical College of Philadelphia, etc. etc. Second edition, revised. 8vo., pp. 768. Philadelphia: Blanchard & Lea. 1859.

A SECOND edition of this work having been called for, the author has faithfully performed his duty in giving it a complete revision. Among the more important alterations, we have noticed the addition of a chapter on Addison's Disease, and a modification of that upon the affections of the chest; while the article on Bright's Disease has been rewritten. Besides these, many additions have been made throughout the whole work, which will thus serve to render it more acceptable, in every respect, to the practitioner. The student who has had the good fortune to attend the admirable lectures of the author, will welcome the present edition as a useful guide in his professional pursuits; while he who is about to enter upon his pupilage, will seek it as a reliable text-book, written in so lucid a style as to be readily comprehended by the dullest intellect.

